# Relational practices for meaningful inclusion in health research: Results of a deliberative dialogue study

**DOI:** 10.1111/hex.13865

**Published:** 2023-09-25

**Authors:** Katrina Plamondon, Davina Banner, Miranda A. Cary, Melissa Faulkner, Heather Gainforth, Kiranpreet Ghag, Alison Hoens, Anne Huisken, Damanpreet K. Kandola, Shaheer Khan, Aline Silveira Silva, Nelly Oelke, Ashmita Rai, Kimberly Strain, Kathryn M. Sibley, Ursula Wick

**Affiliations:** ^1^ Faculty of Health and Social Development School of Nursing, University of British Columbia Kelowna British Columbia Canada; ^2^ Faculty of Human and Health Sciences School of Nursing, University of Northern British Columbia Prince George British Columbia Canada; ^3^ Research and Knowledge Translation in Long Term Care Vancouver Island Health Victoria British Columbia Canada; ^4^ Faculty of Health and Social Development School of Health and Exercise Sciences, University of British Columbia Kelowna British Columbia Canada; ^5^ Department of Physical Therapy University of British Columbia Vancouver British Columbia Canada; ^6^ Rural Coordination Centre of BC Vancouver British Columbia Canada; ^7^ BC SUPPORT Unit Fraser Centre Abbotsford British Columbia Canada; ^8^ Department of Community Health Sciences University of Manitoba Winnipeg Manitoba Canada; ^9^ Knowledge Translation George and Fay Yee Centre for Healthcare Innovation, Rady Faculty of Health Sciences Winnipeg Manitoba Canada; ^10^ Facets Holistic Self‐Discovery Toronto Ontario Canada

**Keywords:** co‐production, equity, knowledge mobilization, knowledge translation, research partnerships, research practices, research systems

## Abstract

**Introduction:**

The importance of including people affected by research (e.g., community members, citizens or patient partners) is increasingly recognized across the breadth of institutions involved in connecting research with action. Yet, the increasing rhetoric of inclusion remains situated in research systems that tend to reward traditional dissemination and uphold power dynamics in ways that centre particular (privileged) voices over others. In research explicitly interested in doing research with those most affected by the issue or outcomes, research teams need to know how to advance meaningful inclusion. This study focused on listening to voices often excluded from research processes to understand what meaningful inclusion looks and feels like, and asked what contributes to being or feeling tokenized.

**Methods:**

In this deliberative dialogue study, 16 participants with experience of navigating social exclusions and contributing to research activities reflected on what makes for meaningful experiences of inclusion. Using a co‐production approach, with a diversely representative research team of 15 that included patient and community partners, we used critically reflective dialogue to guide an inclusive process to study design and implementation, from conceptualization of research questions through to writing.

**Results:**

We heard that: research practices, partnerships and systems all contribute to experiences of inclusion or exclusion; the insufficiency or absence of standards for accountability amplifies the experience of exclusion; and inclusive practices require intention, planning, reflection and resources.

**Conclusions:**

We offer evidence‐informed recommendations for the deeply relational work and practices for inclusivity, focused on promising practices for cultivating welcoming systems, spaces and relationships.

**Patient or Public Contribution:**

This work reflects a co‐production approach, where people who use and are affected by research results actively partnered in the research process, including study design, data‐generating activities, analysis and interpretation, and writing. Several of these partners are authors of this manuscript.

## 1 INTRODUCTION

Recognition of the pervasive contexts of inequities, including those amplified and widened by the coronavirus disease 2019 (COVID‐19) pandemic,^1^ has fuelled urgency for public institutions, including universities, to advance social justice and inclusion. However, normative practices and assumptions in which research unfolds are deeply rooted in structures of exclusion.^2^, ^3^, ^4^ Such structures are revealed even in the definition of inclusion as ‘the practice or policy of providing equal access to opportunities and resources for people who might otherwise be excluded’.^5^ Inclusion efforts striving for *equal access*, however, do not necessarily equate to *equal participation or influence*; rather, they can ignore contexts of inequities and their causes. They also risk overlooking social systems of power that lead to more frequent, comfortable and authentic experiences of inclusion for some, and more frequent experiences of exclusion and tokenism (Tokenism is defined as *including a voice in a project, but mostly ignoring it*. A useful discussion of tokenism is available here: https://blogs.ubc.ca/imhablog/2021/10/13/tokenism-seeing-it-fixing-it-perspectives-from-patient-partners/) for others.

In the context of health research, considerations of participation and inclusion are central to its potential impact and usefulness. Increasingly referred to as knowledge mobilization,^6^ knowledge translation is a dynamic and iterative process of connecting knowledge generation with implementing health research evidence.^7^ *Integrated* knowledge translation (IKT) involves doing research *with* people who use or are affected by it,^8^ often working toward a collective goal together to generate responsive insights, knowledge or solutions.^9^, ^10^, ^11^ While complex, with many different definitions used to convey different characteristics or features,^12^ IKT emphasizes shared decision‐making. IKT's emphasis on *co‐production* ^13^ therefore inherently involves including people outside of academia in processes of connecting knowledge with action. Strategies for ‘engagement’ are often proposed to generate evidence‐based, responsive solutions that can mitigate tokenism^9^, ^10^, ^11^; however, absent of specific equity‐ and evidence‐informed knowledge, skills and practices of inclusion, IKT can reinforce systems of inequity and exclusion.^14^ Yet, even in a review of literature on research partnerships focused on using consensus‐building methods, little research or attention to practices of meaningful inclusion could be found in the literature.^15^

As part of a larger project exploring consensus methods in IKT, our research team was interested in how people describe and what contributes to meaningful experiences of inclusion, asking: *For people who navigate social exclusions, what are their experiences with participating in IKT‐related committees, partnerships, workshops or other events aimed at collective decision‐making? What do people believe is important for leaders, facilitators or organizers of IKT to cultivate inclusion?*

## 2 METHODS

Co‐production is a ‘model of collaborative research that explicitly responds to knowledge user needs in order to produce research findings that are useful, useable, and used’ and is characterized by shared decision‐making throughout processes connecting research with action.^13^ Our process, from conceptualization, team building, to study design and writing, invited an evolving and iterative collaboration among a diversity of people and roles, including patient partners, community members, knowledge users, researchers and people most affected by the research. The team was intentionally inclusive of people who themselves navigate intersecting social exclusions. We worked collaboratively to conceptualize, design and implement the research, along with creating spaces for partnered analysis, interpretation and reporting of the study data. Our process was guided by principles for promoting equity in research and knowledge translation,^16^ operationalized through a variety of mechanisms and practices, including holding frequent dialogue‐based meetings scheduled in ways that made it possible for people to participate.

This co‐production study was grounded in the dialogic and transformative pedagogies of Shor and Freire,^17^ Feire^18^ and hooks^19^ to guide deliberative dialogue—a method for co‐producing knowledge(s) and responses to complex social issues and problems.^20^ This method uses relational facilitation approaches in a workshop‐style setting to invite mutual understanding among a diversity of perspectives,^21^, ^22^, ^23^ typically engaging people in advance by providing preparatory knowledge synthesis and questions. They are future‐facing and creative, convening people in the spirit of learning around a topic of key importance and relevance to themselves and/or their work.

In this study, preparatory materials included a brief plain language summary of scoping review findings,^15^ questions to guide reflection and an invitation to use collage‐making to express their understanding of *inclusion*.^24^, ^25^ Using art‐making as a predialogue reflective activity was grounded in both literature on the value of creative practices in research co‐production^26^ and our previous experience with this method as a means of generating shared insights and sparking reflective dialogue around complex topics. A series of 2‐h Zoom‐based virtual dialogues were held over a 2‐week period in March 2021, with two follow‐up public knowledge dissemination events 6 weeks later. Dialogues were guided by an expert lead facilitator and supported by two note‐takers, a designated support person, and a logistic facilitator. Dialogues centred around an intentionally open and exploratory question, asking *what does inclusion look and feel like?* Participants were invited to share their collages, which served to spark dialogue and invite others' reflective responses. As a team of people who also navigate intersecting social exclusions, the research team were active listeners and contributors.

Dialogues were audio‐recorded. Data sets included audio recordings, note‐takers' notes, Zoom chat box contributions, collages shared by participants and facilitators' notes. Data were analysed in NVivo 12, balancing analytical lenses to look for deep contextualization while attending to both the richness and silences in data.^27^ Preliminary analysis categorized and summarized large volumes of data (by A. H.2 and K. P.), which were then offered in a report of results circulated to participants for comment and shared in follow‐up dialogues open to all members of the research team and all participants for validation and discussion about practice implications. Analysis was further nuanced by bringing emergent findings forward to critically reflective dialogues with the broader research team,^27^ where those responsible for the time‐intensive work of moving through the data could listen for direction, and return to the categorizing‐summarizing process in response.

Our commitments to co‐production and critical pedagogy were extended through this iterative and critically reflective process to support analysis, interpretation and writing. Critically reflective inquiry involves the ‘conscious interrogation of the social, cultural, and political contexts of learning’,^28^ situating dialogue in the context of people's day‐to‐day lives and practices while unpacking assumptions and considering others' perspectives. Over the course of the study, and for more than a year after the last deliberative dialogue, all 16 members of our research team contributed to learning together through deliberation about the implications of what we heard from study participants. Meetings were generally facilitated by K. P., using reflexive and relational practices for supporting dialogue.^22^ Honouring the time, skills and expertise of team members, each meeting included an overview of progress. Meeting times, duration and structures were designed to respond to the diverse accessibility needs of each member of our team, accounting for a range of factors influencing opportunities to contribute (e.g., needs related to health and wellness, childcare, geography, time zones). We allowed sufficient time to begin with a check‐in, respond to emergent issues, plan for next steps and close with a round of reflective comments and wishes from each contributor. We found that 1.5–2 h were needed for each meeting.

### 2.1 Participants and recruitment

We sought participants who self‐identified as: (a) navigating social exclusions related to race, ethnicity, gender, ableness, healthfulness, Indigeneity, sexual orientation, or other intersecting identities; and (b) had IKT‐related experience(s) as partners on research projects, advisory teams, panels and committees. People were recruited within Canada by email, newsletters and social media. Each participant was invited to complete an anonymous ‘perspectives’ survey (see Table 1). If people chose to create a collage, they were invited to upload their content electronically and complete an art release consent. Participants were offered a $50 honorarium for their time, which was estimated at between 2 and 4 h.

**Table 1:** Inclusion criteria and perspective survey.

To participate in this study, participants needed to:	
Be 18 years of age or older Be able to contribute to dialogue in English, either independently or with the support of a translator or interpreter Have work (volunteer or paid) experience participating in research partnerships (teams, committee, advisory groups, community) Identify with at least one group who navigate social exclusion(s) Be able and willing to provide informed consent	
Perspectives gathered from participants (before dialogue)	
How old are you?	18–25; 25–34; 35–44; 45–54; 55–64; 65–74; 75+
Which gender do you most identify with?	Man; woman; transgender man; transgender woman; gender non‐conforming; something not listed‐specify; prefer not to answer
Do you identify with any of the following? (check all that apply)	Belonging to a visible minority or racialized group Belonging to an excluded socioeconomic group (e.g., ‘blue‐collar’ or working class) Living with a visible or invisible disability) Lesbian, gay, transgender, bisexual, queer, questioning, two‐spirited, non‐cisgender or other‐than‐heteronormative First Nations, Métis, Inuit or Indigenous Having a ‘patient perspective’ by virtue of your experiences navigating healthcare systems or volunteering with a patient‐oriented research or patient‐oriented healthcare advisory group, such as the Patient Voices Network Having lived experience of navigating housing insecurity or houselessness Having lived experience of navigating substance use as a family or as an individual Belonging to any other group that has experienced systematic exclusion or unearned disadvantage in a setting where other groups benefit from unearned advantage (if you are comfortable doing so, please describe)
What kinds of research committees, partnerships, or advisory groups or decision‐making ‘tables’ have you participated in before?	Open‐ended
Why were you invited to participate (e.g., was it to bring a participant perspective)?	Open‐ended
Were you compensated for your contribution?	Yes; no; both
In this process, did you contribute as a volunteer?	Yes; no
In this process, did you contribute as a paid employee of an organization?	Yes; no

### 2.2 Research ethics

This study underwent a harmonized ethical review by the University of British Columbia Behavioural Research Ethics Board (H18‐03416). All participants provided written or verbal informed consent and consent was reconfirmed in dialogues. Among efforts to ensure relational accountability in our process were attention to language and accessibility (e.g., visual accessibility of study materials), as well as providing dedicated, individualized support to participants in advance of and through follow‐up dialogues.

## 3 RESULTS

**Figure 1:**
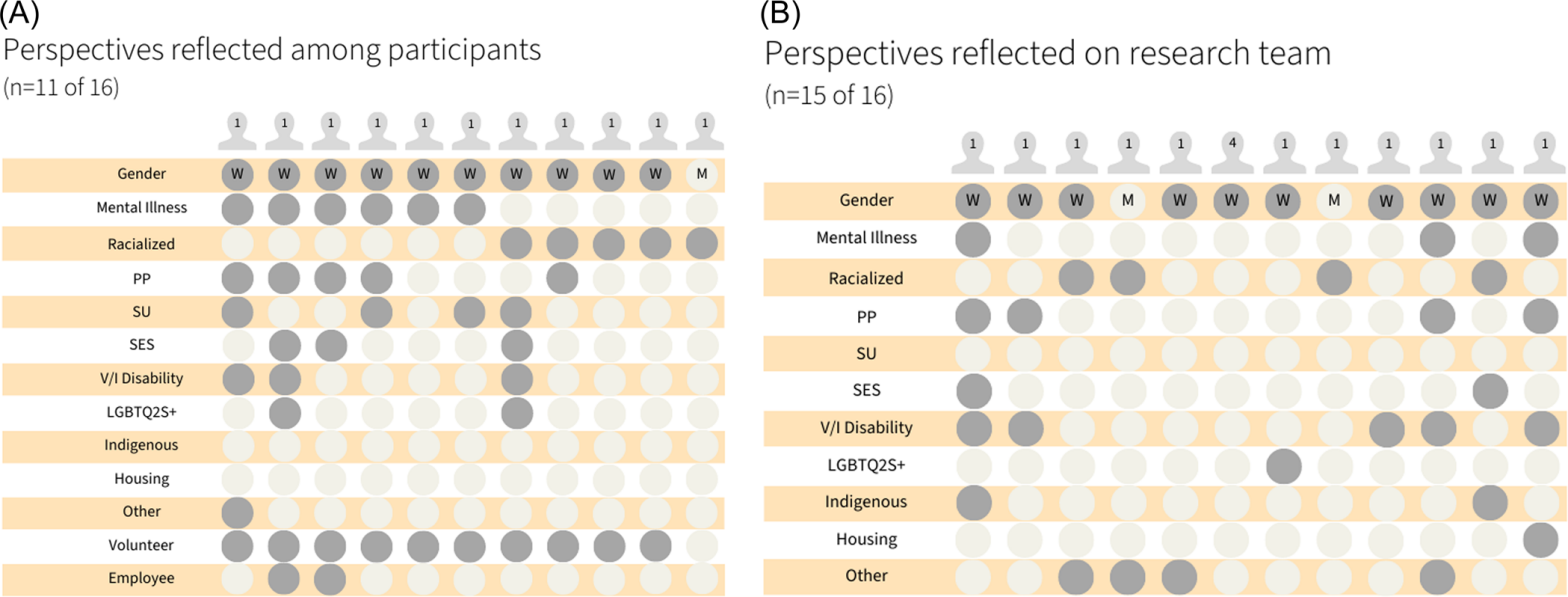
(A) Intersecting perspectives reflected among participants, with each icon representing one person's self‐reported positionalities. (B) Intersecting perspectives reflected among research team members, with each icon representing one person's self‐reported positionalities. Self‐reported perspectives are highlighted with a grey dot. Housing, experience navigating housing insecurity or homelessness; Indigenous, identify as First Nations, Métis or Inuit; LGQBT2S+, identify as lesbian, gay, queer, bisexual, trans and two spirit (or other); M, identify as man (no others reported); mental illness, experience navigating care for mental illness; other, experience with any other social exclusion, as defined by the person; PP, patient perspective, patient partner in integrated knowledge translation or related activities; racialized, identify as a visible minority; SES, experience in an excluded socioeconomic group; SU, experience with or related to substance use; V/I disability, experience living with a visible or invisible disability; W, identify as woman.

A total of 16 participants (4–10 per event) from across Canada contributed to dialogues, in addition to five research team members. All research team members participated in the collaborative, dialogue‐based process of analysis and interpretation. Of the 11 participants and 15 research team members who completed the ‘perspectives survey’, most self‐described navigating multiple social exclusions. In Figure 1A,B, we show the diversity of perspectives reflected among participants (Figure 1A) and research team members (Figure 1B), using highlighted dots to highlight each person's unique intersectionality of experiences, positionalities and identities. All study participants reported either being a ‘patient partner’ or bringing a patient perspective, or representing a specific community partner in their IKT contributions. Most experiences were as volunteers; however, two reported experiences in paid roles. Unless otherwise noted, data reflect an intersectionality of ‘patient’ or ‘community’ partner perspectives that, because of our ethical obligations and the dialogic nature of the data, cannot be reduced to a singular lens.

Dialogues generated many insights into the assumptions, practices and structures that contribute to meaningful inclusion. Below, we organize dialogue results by how *welcome* in systems, spaces and relationships cultivates meaningful experiences of inclusion (Table 2).

**Table 2:** Themes and subthemes.

Main theme	Subthemes
Welcoming systems	Being self‐reflective, aware of and responding to power dynamics Centring values, policies and norms to enable inclusion
Welcoming spaces	Setting a table of welcome, and extending it over time Facilitating the logistical and practical supports
Welcoming relationships	Valuing many perspectives, expertise(s) and experiences Honouring relationships and their complexity

### 3.1 Welcoming systems

In all three dialogues, participants' reflections began with a focus on research systems, which were described as the structures, institutions, people, norms and values that enable research. Participants described these systems as rooted in colonialism and white supremacy that serve as complex determinants of the direction, standards and norms of research.

Deep appreciation for the complexity of power dynamics arose repeatedly and participants noted the importance of cultivating and nurturing relational practices and inclusion, including recognizing and reflecting on positionality, power, and worldview. Despite valuing these practices, participants frequently reflected on how ‘inclusion’ is often spoken about rhetorically, while remaining superficial or reinforcing dominant colonial frameworks. One person commented,

I really struggle with it when people say diversity and inclusivity because you can still have a colonized mind and colonial thinking.

Participants commented on the tendency for positions of influence to be occupied by ‘*experts*’, reproducing and celebrating privilege for already privileged groups. In doing this, participants drew focus on potential lack of alignment between aspirational practices and statements to the enactment and operationalisation of those strategies through practice. One participant reflected,

…the number of publications or how much you've worked in a field means that you get more inclusion, or more opportunity to talk, or write, or get involved with the project, so that differential value assigned gives a different opportunity to be heard or have space to speak.

Participants understood the reproduction of privilege as one‐way research systems themselves create and reinforce inequities.

Participants critiqued the insufficiency of accountability mechanisms within research systems. They described a paucity of standards, principles, practices or expectations to report on research practices after research ethics review, and problematized the reliance on researchers' self‐report. For example, some participants described the challenges faced by people in informal IKT ‘partner’ roles who, unlike research participants, do not provide formal consent or have access to an ethics board for support. Nor are they hired by a university, so do not have access to the protections or oversight they might receive as an employee. For many, this resulted in liminal and uncertain engagement spaces, with little protections being afforded.

People also problematized a general tolerance of *rhetorical* inclusion with indemnity, poignantly explained by one participant who pointed to tokenized naming of ‘inclusion’ principles:

Academia set up this committee for diversity, inclusion, equity … nobody is questioning it because the Doctor is very influential and she started this committee, … It feels like [in these situations] I am the informant at the grassroots level. There are two guiding principles for meaningful engagement with people with [lived experience]. The principles are imbedded with research documents, and it's like tokenizing us … but just because we put these principles on the table, everything is assumed to be okay.

Participants lamented the absence of mechanisms for reporting or evaluating their IKT‐related experiences. Participants desired learning‐focused and nonpunitive feedback systems for critical reflection that could support all people involved in IKT.

### 3.2 Centring values, policies and norms to enable inclusion

Extending from these calls for accountability were reflections on values, policies and norms that could enable inclusion. Several participants expressed frustration with institutions and researchers' tendencies to overstate progress and overvalue mere *appearances* of inclusion. Participants believed academic institutions could place greater value on inclusion by embedding safe, accessible third parties, such as a neutral Ombudsperson. They also called for greater value to be placed on the time, trust and relationships required for meaningful inclusion. For example, one participant who identified as Indigenous described the *reason* for meaningful engagement as her life‐long relationship with, and accountability to, community and future generations—wherein a lifetime of relational accountability could not be leveraged for a short‐term project.

Participants also reflected on how standards of worthiness in academic institutions place the greatest value on individual prestige, funding and productivity. These entrenched norms were described as ways in which institutions work against meaningful inclusion. In all dialogues, participants reported frustration with the lack of attention, incentive, value and funding afforded to the time and relational work of building trusting relationships. We also heard compassion for the barriers researchers face in practicing more inclusive research practices, by virtue, for example, of funding requirements or norms that undervalue the costs and time required to cultivate inclusion. Given the competitiveness of research funding, researchers who wish to cultivate welcome and inclusion must push against strong and deeply entrenched systems and norms that often do not value or *directly oppose* such practices.

### 3.3 Welcoming spaces

Dialogues were rich with insights on what it takes to create a feeling of welcome and inclusion in research spaces, particularly emphasizing this feeling as having to do with interpersonal interactions.

We heard that inclusion did not have to be a grand gesture. People shared the importance of humility, reflection and checking unconscious bias and assumptions as critical practices for inclusion. Further, participants suggested that taken‐for‐granted assumptions from dominant, able‐bodied, cis‐gendered, privileged groups often place heavy burdens on people who are already navigating multiple systems of disadvantage. They asserted that partnering and relational practices shape the degree of inclusivity possible in any kind of research process. Accessibility considerations were extended to the taken‐for‐granted assumptions about meeting expectations and spaces that serve as consistent barriers to meaningful inclusion. Participants reflected that these assumptions translate into unfair burdens in, for example, expectations of preparatory reading,

…for me it means translating first in my head then [back to English] so it takes twice as long … creates a lot of extra work that you don't necessarily understand…

Above all we heard that all researchers, institutions and systems can cultivate, learn and apply more inclusive research practices.

Participants frequently spoke to the importance and impact of practical engagement supports on research and IKT relationships, noting the need for teams to be attentive, reflective and responsive to diverse needs. For example, we heard from people who navigate the world with an invisible disability that their ability to participate in research requires ongoing dedicated support, noting that these costs were often seen as ‘too much’ or overtly excluded from research budgets. When budgets are not adequately allocated for services, for example, participants suggested that costs are generally passed off like a ‘hot potato’. This passing off of costs was observed as positioning particular departments (e.g., equity offices or accessibility programs) as solely responsible for making spaces inclusive. Reflecting on a participatory workshop specific to including deaf people, one participant described the consequences of inadequate budgeting for sufficient American Sign Language interpreters,

When it came time to break into smaller groups, many people in the room were upset because the limitation of having only have 2 deaf interpreters [for 20 people] meant that instead of mix and mingling with us and share their views with everyone else [in the room] they were relegated to communicating amongst themselves, we the non‐disabled in the room … missed their input and their conversation because they [the organizers] did not stop to think that with the number of people that were there and the number of tables they should have had two interpreters per table to allow the proper mix and mingling of the deaf community, inclusion requires intentionality…

Participants also reflected on how compensation norms and practices shape experiences of inclusion, noting that vast salary inequities or expectations of volunteerism create differential costs of participation. One participant described her experience asking for compensation for a position on an advisory committee,

A lot of the advisory groups I've been part of, I've done for free … . So I decided to ask for compensation for the chair role I had … you would have thought that I asked for the moon! … they ‘so and so’, who is a big wig in research, ‘they didn't ask for an honorarium!’… And I was really made to feel it and every time I asked for my cheque it was delayed or it took forever, they made me work for it. I loved everything else about that job, but it made me so mad!

Participants recognized that not all costs or compensations are financial, but can also be social, emotional, cultural or relational. One person reflected on the emotional–cultural–relational costs of expectations put on them, and others in their community, to ‘show up’ in service of ‘equity, diversity and inclusion’,

Whenever there is a call for participation, call for diversity … I usually feel a natural responsibility to be there because, if there is no one there from [my identity group] … it looks like nobody wants to be there. It is usually not paid … [but] everything I do in my spare time needs to be directed to me making some kind of money … meetings are set up on the free time for people who have the time for them … usually at lunch then there is no food, you gotta find your way to feed yourself … food is equity piece of saying I am giving you my time, knowledge, time means I won't be able to work or make money, I'm not asking for a free meal I'm asking for an equity piece for my contribution … my time and my knowledge is what I value the most and it's what I have to offer…

From participants' perspectives, creating welcoming spaces over time required attention and responsiveness to dynamic issues of power and equity that manifest through practical, logistic and often taken‐for‐granted aspects of IKT.

### 3.4 Welcoming relationships

Many reflections about what contributed to meaningful experiences of inclusion connected interpersonal interactions and intrapersonal characteristics, practices and skills of people in positions of influence (e.g., researchers, decision‐makers, academic leaders). This included a desire for authentic relationships, commitment to self‐awareness and self‐reflection and intentional efforts to enact policies and practices that establish and sustain inclusion.

In one dialogue, participants explored the rhetoric of equity, diversity, and inclusion (EDI) as often feeling superficial. They described the effects of this superficiality as generating burden for those ‘filling a seat’.

EDI is something I hear way too often … when I hear it […] it really doesn't mean anything to me … it seems like people are looking to me because I fill the diversity quota and just for being in the room it already feels that I am also filling that spot of diversity and equity…

The conversation continued with another reflection about how simply being present does not equate to meaningful inclusion,

…I usually have the responsibility to make sure other folks who do not have English as a first language, they are understanding what's happening in the room. It usually comes down to me to make sure that people understand what is happening and I feel that that's something that is given to me because of the fact that I can speak [English] a little better than other folks who are at that table.

Others also spoke about being tokenized or overtaxed to ‘represent’ whole groups of society. In their experience, this could lead to feeling more isolated:

…I have been tokenized as ‘the disabled person’, I don't have any experience with being a person with low vision or deaf but I have experience with chronic pain issues and mental health issues and feeling like I have to think of all disabled people's issues and accommodations when I don't have those experiences…

She also worried that she had satisfied the quota and that the research team would not be willing to provide training or opportunities to others with different experiences, saying ‘they'd be like “Oh … we can't find another disabled person with lived experience”.’ She found herself in a difficult position of over‐representing persons living with disabilities and was hesitant to speak up about her experience or skill set.

Another person added the nuance of meeting ‘criteria’ for inclusion, while simultaneously being devalued. Developing inclusive partnerships, they argued, requires a high level of self‐awareness, integrity and intentionality from researchers in particular, who are often positioned in leadership roles, and from all people engaged in research teams, committees or partnerships.

One participant explained how inclusive partnerships require that people in positions of influence are aware enough of themselves to be able to set the tone for how others are welcomed and valued in a research team. He offered an example of the differential value placed on different people:

We have one hour meeting you can talk for 35 minutes. Sometimes [researchers are] biased and think that someone is very knowledgeable, and he can talk more about it [the topic] and someone [else on the team‐‐he has literally no papers in this research field and he may not have many things to say. We will just assign him 10 minutes for the other 15 minutes because he has more papers and his [expertise] is more related to this topic.

This example indicated the importance of holding all experiences and knowledge with respect and value. These examples highlighted the importance of people's willingness to be self‐aware and open to learning.

In every dialogue, we heard experiences of inclusion involve relational and brave practices. Participants reflected on the subtle ways in which power hierarchies in research relationships are established at the start of meetings or new initiatives. Further, participants were frustrated at repeat demands to repeatedly tell their ‘diversity’ story or justify their participation in research or research systems,

In my experience this means that … frustration when you leave a meeting and you did your contribution to that inclusion or equity and the next time you come to a meeting it feels like you're starting all over again … there's just some days that you don't want to have that conversation again…

Participants agreed that cultivating meaningful inclusion in either research systems or practices requires relational efforts that challenge tendencies for ‘equity’ checkboxes that rely on linearity.

Participants emphasized the complexity of these relationships, and the skills needed to respect the nonlinear nature of relationships. They also pointed to the importance of having the kinds of equity‐responsive skills needed to cultivate reciprocity and dismantle hierarchies. Striving for open and transparent communication about what people want and need from research relationships, and understanding the implications of requests for them to contribute. People shared unease with rushed, superficial, transactional kinds of research relationships that led to feelings of tokenism. As one participant described, ‘inclusivity is nothing without equity…’. Participants described the importance of building relationships from a position of trust, humility, listening and continuous commitment.

## 4 DISCUSSION

This dialogic study deepened our understanding what authentic, meaningful inclusion looks and feels like, illuminating the importance of working across multiple levels of research and research systems to grow collective awareness and capacity. Results of this deliberative dialogue study affirm long‐standing critiques of the insidious and tenacious ways in which social systems and structures serve to perpetuate exclusion, specific to research‐related settings. A rich body of critical scholarship has long called for transformations in social systems, structures and practices of social exclusion, and offers thoughtful, practical guidance for how to do so.^18^, ^29^, ^30^, ^31^ In academic settings, the effects of systemic inequity and exclusion are well documented, for example,^32^, ^33^, ^34^ as academic settings scramble to make public statements to declare their virtue as institutions that uphold EDI,^35^, ^36^ few examples go beyond politically correct rhetoric.

Study participants' reflections on the absence of accountability for inclusion, for example, point to systems‐level gaps—even in the research mechanisms intended to facilitate inclusion. Assumptions, for example, that *intention* itself can lead to inclusion exemplifies the absence of understanding or value placed on complex relational practices needed to challenge systems of exclusion. Study participants here suggested the need for transformational changes in the distribution of power, both in research systems (e.g., research ethics boards and affiliated policies/practices; research committees; research funding; peer review) and research practices (e.g., research team practices; authorship practices; for example,^37^ methodological and study design issues; partnering practices; and research processes).

We also heard that meaningful inclusion is about honouring and upholding dignity in ways that prioritize genuine welcome, embrace humility and listening, and dismantle hierarchies. Planning *places of welcome* was described as central to cultivating an environment of inclusivity, where room is made for many voices. This result resonated with one our research team members who works with people who have spinal cord injuries. They described creating *places of welcome* as involving many practices, from removing physical barriers to participation (e.g., chairs in meeting rooms) to practical barriers to participation (e.g., avoiding early‐morning meetings). Whether for a specific population, like people with spinal cord injury or not, it is clear that creating a place of welcome necessitates budget and time for adequate, responsive compensation and practical support.

Dialogue results emphasize that meaningful inclusion is far more than filling a seat at the table. Several models and tools aim to respond to the notion of inclusion and engagement.^38^, ^39^, ^40^ Arnstein's *Ladder of Participation* described eight ‘rungs’ to represent progressive degrees of less or more meaningful citizen participation, ranging from nonparticipation to degrees of tokenism and degrees of citizen power.^41^ Cited more than 26,000 times since its publication, this visual is immediately recognizable across disciplines, sectors and settings. Despite enthusiastic uptake of the visual model on paper (or screen), Arnstein's critique of systemic social exclusion and call for transformational justice of inclusion is not yet realized. She situated the model in the context of ‘profound inequities and injustices’ that shape the daily lives of ‘have‐nots’, who are often framed by power‐holders as a homogenous and dehumanized group of ‘others’. She argued that ‘participation without redistribution of power is empty and frustrating’ (p. 24) and named racism, paternalism and resistance to power redistribution as major obstacles to meaningful inclusion.^41^ The concerns raised decades ago by Arnstein and many other critical scholars before and after remain compelling.

The dialogues in this study touched on issues that can be situated in organizational cultures of white supremacy, which elevate individualism, perfectionism, urgency and quantity over quality, for example, ^42^, ^43^ research and research systems reflect broader societal intersectionalities, wherein social, cultural and economic positionalities (e.g., race, ethnicity, sex, gender, ableness, Indigeneity, sexual orientation) serve to perpetuate social exclusions. The *effect* of these intersecting positionalities is unearned advantages for some, generating a power dynamic that shapes how decision‐making and communicative power is shared and/or balanced within the research partnership and project.^44^ Despite the popularity of EDI as a buzz phrase, its rhetoric can do more to distract than mobilize systems‐level transformations needed to combat tokenism and foster meaningful engagement.^36^ A rhetoric of EDI might sound good and serve to garner public support and avoid scrutiny, but superficial efforts to simply incorporate diverse bodies into oppressive systems are specious steps toward systems that need to genuinely honour, respect and create space for diversity in perspectives and voice.

Much of the rhetoric of inclusion asks ‘patient’ and ‘community’ partners to be vulnerable and share very intimate details of their lives when they are invited to participate. Not only is this vulnerability unequally demanded it is often asked with haste so that teams don't ‘waste’ too much time on introductions. Even researchers who occupy positions of power and advantage relative to their research partners can be situated vulnerably in research systems. Researchers who use participatory methods or IKT approaches are often pushing against hierarchies and systems of unearned advantage for able‐bodied, cis‐gendered, white, men. Wallerstein et al. argue that

despite its values, participatory research still resides within inequitable research hierarchies. Funding, technical expertise, and institutional resources are overwhelmingly controlled by funding agencies, academic administrators, and (to a lesser extent) researchers. Most research is conducted by majority‐white institutions led by white and other PIs with normative privilege (e.g., male, hearing)^45^

Work undertaken to be inclusive in this environment of ‘privileging privilege’ can be viewed as radical. Challenging systems of privilege is challenging work, requiring deep attentiveness to the mental models and practices that hold those systems in place.^4^ Practicing inclusion invites critical examination of climates of privilege and advantage. Attentiveness to how systems of power create unearned *advantages* is as important, and perhaps more important, than redressing systemic disadvantage.^45^, ^46^ Advancing meaningful inclusion is, therefore, not about requiring disadvantaged people to share their stories and justify their place at a table, but rather, about eliminating the structural advantages that lead to social exclusions. It is the responsibility of *power‐holders* to recognize and remove the features of the environment and relationships that generate inequities.

University systems, broadly, offer little support for researchers to work toward this idea. Academic institutions can do more to recognize the value and realities of more inclusive research processes, which might not conform to traditional metrics of success (e.g., volume of publications vs. quality of relationships or responsiveness of research). Funding agencies could do more to design competitions that allow for sufficient flexibility and time for relational practices and enable more inclusive and flexible compensation options.

Cultivating meaningful inclusion requires practicing integrity in our commitments to transformational change and challenges the way researchers (and others in positions of influence) think and do.^47^ It invites us to shift the way inclusion practices are documented and afforded valued space in publications, how researchers demonstrate their inclusion practices in tenure and promotion packages, in how peer review prioritizes the demonstration of meaningful inclusion, in how funding agencies and institutions recognize and value inclusive practices, and in how researchers and research systems are held to account. Researchers themselves gain from including others in their work and are changed by it. It can shift the way they design, deliver and disseminate research.^47^ Yet, research systems are not set up to make these changes and are not designed around transparency or accountability.

## 5 TOWARD INCLUSIVE RESEARCH SYSTEMS AND ENVIRONMENTS

**Figure 2:**
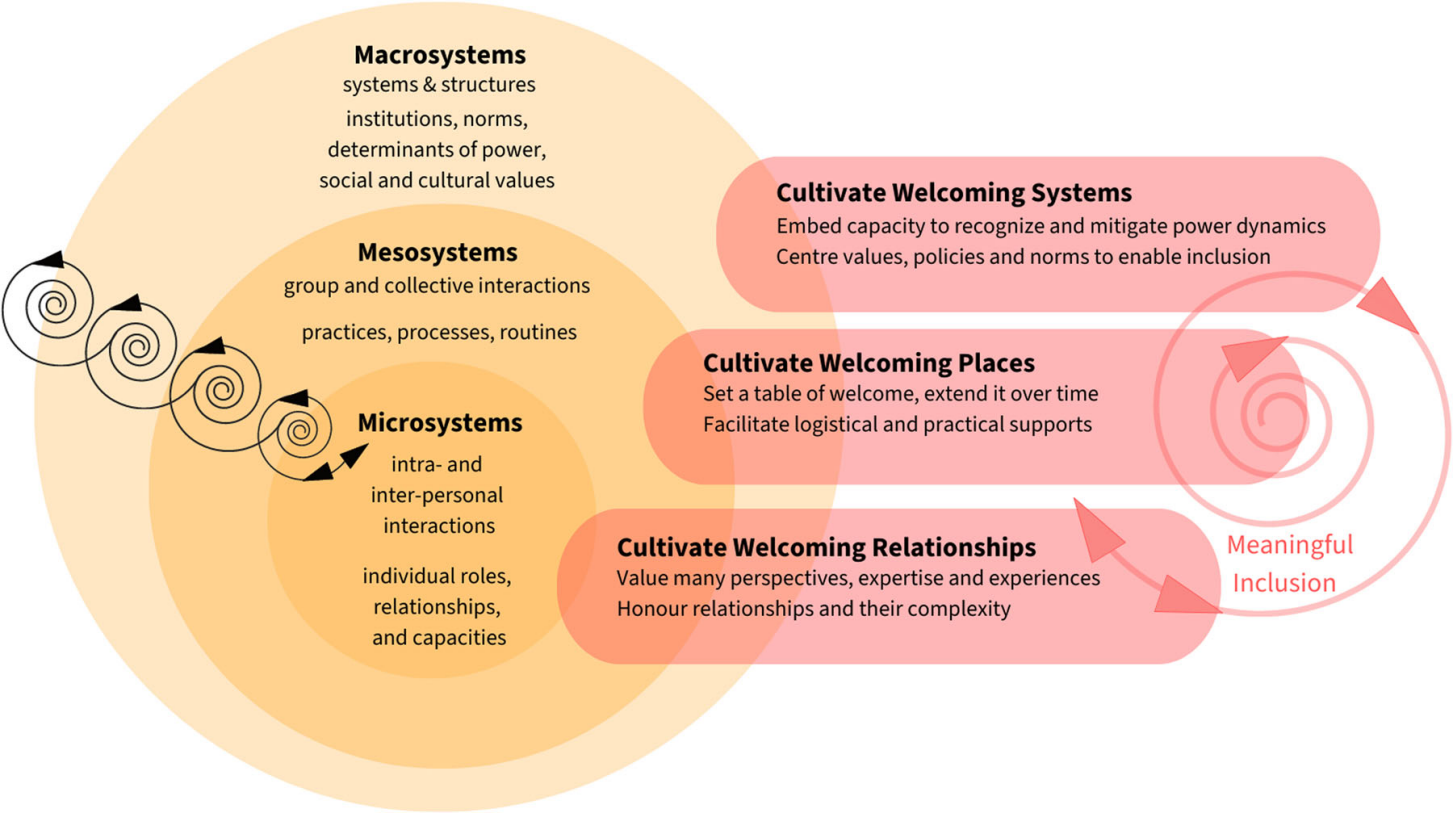
Cultivating welcoming systems, places and relationships from micro‐ through macrolevel of context.

Several specific recommendations arose from our team's critically reflective dialogues on study results and implications. As we worked with and contemplated this data, we found Brofenbrenner's social–ecological model^48^ useful for its emphasis on intrapersonal, interpersonal and systems interactions that can contribute to meaningful experiences of inclusion by nurturing welcome. Below, we offer a working conceptual model and set of promising practices that directly respond to study data and also to the experiences and knowledge(s) of those on our team—particularly those who shared an ‘insider’ perspective. By ‘promising’, we mean that there is both theory and evidence which can support and advance meaningful inclusive practices. Figure 2 shows: (a) the dynamic and interactive nature of macro‐, meso‐, and microlevel of research ecosystems; and (b) promising relational practices that can be extended at each level to cultivate meaningful inclusion. We use the image of a progressive spiral spanning each of the levels of ecosystems, and the kinds of welcoming practices at each level, to demonstrate the relational connections between each. Table 3 provides more detail on how to enact each of these relational practices.

**Table 3:** Promising relational practices for moving toward meaningful inclusion across research ecosystems.

Ecosystems Level^a^	Relational Practices	How to move toward meaningful inclusion
*Macrosystems* Broad, higher level social systems and structures give shape to institutions, norms and determine the distribution of power. Includes social and cultural values, particularly those privileged in society Governments, funding agencies, universities (administration, leadership, research ethics boards) and any others who play a role in setting policy directions affecting others across the ecosystem are responsible for cultivating inclusion at this level	Create policy environments to enable active inclusion	Accept responsibility for cultivating inclusion through systems Leverage positions of authority and influence to advance more inclusive systems and structures Increase alignment between aspirational or strategic statements about equity and inclusion and the operational environment in which those strategic visions exist Develop and enact policies to provide researchers and research‐related committees with guidance about equitable remuneration for contributions Endorse inclusive practices and declare them as worthy across other ecological levels
	Integrate equity considerations in systems, structures and organizations	Acknowledge the role of systems and structures in shaping conditions of unearned privilege and inequity Build processes and budgets that provide adequate and equitable resources to enable meaningful contribution Dedicate resources and supports that enable inclusive practices by those within your organization Create mechanisms for relational accountability with the establishment of a position that is responsible for responding to the needs and concerns of people impacted by research through, for example, an Ombudsperson Prioritize inclusion in all research and IKT efforts including (but not limited to): publication policies; peer review policies; grant review policies; funding mechanisms and policies; research ethics processes and systems; strategic planning and resourcing related to training and education; hiring policies and practices; and committee composition (e.g., terms of reference)
	Challenge norms and assumptions that inhibit meaningful inclusion	Adopt flexible practices and policies to meet the needs of all participants
		Analyse and work toward more equitable policies and norms that shape ideas about what is considered a ‘good’ way of working (i.e., equitable practices are more promising than those that entrench inequities)
		Invest in capacity‐building opportunities for people within research systems to: ○ Practice cultural humility and safety ○ Ensure that participation does not create undue hardship ○ Encourage dialogue to challenge assumptions about ‘good’ practices for inclusion ○ Create transparent and responsive processes to identify and respond to individual needs ○ Collectively articulate equity needs and goals, drawing upon resources to identify, assess, and redistribute power (e.g., establish equity‐centred governance practices)
		Practice honouring many contributions and voices in ways that uphold/recognize their worthiness, the expertise of experience and tacit knowledge
*Mesosystems* Faculty, department or unit‐level systems that implement and extend mid‐level policies, procedures and norms. Includes standards and expectations that shape research interactions, meetings, processes and routines shared by collectives or groups Researchers, teams within funding agencies, peer review panels and others involved in IKT and related activities and research processes as part of their paid work are responsible for cultivating inclusion at this level	Cultivate places of welcome	Recognize creating experiences of welcome as sophisticated and relational work, requiring specific skills and competencies Think about inclusion like you are setting a table and want everyone who joins to feel like they are welcome, feel comfortable and able to participate and contribute Invite people to co‐design the *where* and *when* of meetings Welcome people with warmth and a genuine desire to understand their perspectives Cultivate belonging in the ways in which people gather and are introduced
	Commit to extending welcome over time	Amplify the value placed on the experience of welcome by building capacity for its practice Integrate budget, time and resources to make welcome possible Offer gifts as gifts (not compensation) Recognize honoraria as reciprocity and compensation, rather than gifts
*Microsystems* Immediate environments that shape research relationships, including intra‐ and interpersonal interactions that shape who and how a person interacts with others Researchers, leaders and anyone involved in IKT or related activities who is in a position of privilege or power are responsible for cultivating inclusion at this level	Practice reflexive, responsive self‐awareness	Accept responsibility for nurturing inclusion Adopt a consistent practice of reflection on positionality and intersectionality Assume there are many things you do not know, including not fulling knowing how people may be impacted by your efforts to use or do research Assume that you navigate the world from a different worldview and set of advantages and disadvantages (e.g., social, physical, emotional, geographical, financial) than others; seek to understand others' worldviews Critically reflect on how your assumptions and advantages shape the way you think about engaging others in using or doing research
	Build capacity and agency for relational practice	Amplify the importance of self‐awareness and attention to positionality: ○ Speak about reflexive practices in academic meetings, assessment environments and peer‐review settings ○ Develop relational practice skills amongst your team, trainees and students ○ Write about your team's commitment to reflexive practices in your publications, using supplemental files if needed ○ Build expectations for reflexive practices into mentoring plans and peer‐review practices ○ Demonstrate and model the value of reflexive practices by including statements about what you are doing, why it matters and observed impacts or actions taken in teaching philosophy statements, annual reports or other demonstrations of academic contribution
		Practice deep attentiveness and listening for direction
		Demonstrate the interest and value you hold for others' perspectives
		Practice humility by adopting a position of curiosity and learning (rather than knowing)
		Make time to develop relationships and resist the pressure to rush
		Accept and make room for the dynamic, nonlinear nature of relationships
		Build relationships up and out, networking broadly to reach other individuals, particularly those voices that are not usually heard
		Spend time with and in communities to learn and respond to contexts, needs and hopes as they understand them
	Build capacity and agency for inclusive interpersonal interactions	Build collective capacity (knowledge and skills) to recognize, understand and productively mitigate power so that it is distributed in ways that promote inclusion (e.g., take training programs; learn together through communities of practice; and create opportunities in courses, seminars, professional development opportunities or meetings) Engage in exploratory dialogue with others to begin to identify and respond to the things you do not know Ask what people would like their contributions to be Inquire about what a good outcome or experience would be Find out what resources/supports are needed to enable meaningful contributions Invite many voices to the table; create multiple tables if that is what works for voices to be heard Consider positioning research in service and responsiveness

^a^ Levels of social–ecological systems are informed by Bronfenbrenner's (2005) Ecological Systems Theory.

### 5.1 Strengths and limitations

A unique strength of this study was the use of dialogue‐based methods for generating, analysing and interpreting data. Distinct from extractive, one‐directional approaches to qualitative analysis,^27^ we created a rigorous process that balanced integrity in methodology with a collaborative process that enriched contemplation of research findings in the context of real‐world experiences. Infusing the analysis and interpretation process with critically reflective dialogue among the research team ensured a diversity of perspectives was always present. As a result, we were able to dive into the data with nuanced, rich and applied lenses, exploring both meanings and implications in ways that allowed practical recommendations to emerge. Importantly, attentiveness to reflexive and relational practices in the process invited members of the research team to consider direct, immediate implications in *their own practices*. Our co‐production approach was, in this way, a transformative, challenging assumption and raised awareness of how each of us can more authentically practice inclusion.

While we do not assert our recommendations are in any way exhaustive or universally representative, the dialogue‐based processes involved in this study means they are grounded in a deep respect for a diversity of knowledge (practice‐based, experiential, cultural, organizational, systems, disciplinary, among others), experiences, and perspectives. Despite efforts to practice inclusivity in our recruitment, we did not reach all groups who experience social exclusions. Perhaps because of the topical emphasis on IKT, or perhaps a reflection of systems of privilege that may serve to make issues of inclusion less of a pressing issue for men, the vast majority of research team and participants self‐identified as women. Members of the research team who had expertise in research analysis were tasked with working through raw data from dialogues and preparing summaries for participants to review and validate. While this places the time‐intensive work of synthesis on people who were paid to do so, it means that the interpretation of our data is influenced by their lens and their positionality. We mitigated the potential for a heavy ‘researcher gaze’ on our data by creating an inclusive, critically reflective dialogue‐based process of ‘writing through’ our results.

Another consideration in our process was the use of a virtual environment for dialogue. This was necessary because of the pandemic‐related public health restrictions faced at the time and could have played a role in the experience, power dynamics and facilitation of the dialogues. The virtual environment may have appealed specifically to people who were comfortable in such an environment and may have limited the participation of some people with less familiarity or access to computers. While we cannot know for certain how the virtual space may have played a facilitating or inhibiting role on experiences of inclusion, we heard a high degree of satisfaction from participants and were affirmed by their expressions of interest in follow‐up activities. When we asked participants about who needs to hear about the results of this study, the overwhelming response was to share these results widely across diverse settings and groups. For example, one participant responded, ‘Everyone! Everywhere! These are such important things to say, and I hope you will share it widely and keep inviting us to be part of the conversation!’

## 6 CONCLUSION

Paulo Freire, whose work was influential in the design of this study, spoke in existential ways about the importance of inclusive societies. He argued that ‘dialogue phenomenizes and historicizes essential human intersubjectivity; it is relational, and, in it, no one has absolute initiative. Dialogue participants admire the same world; they move away from it and coincide with it; in it they are placed and opposed … dialogue is not a historical product; it is history itself’.^18^ As researchers or leaders in research systems, the world is facing a moment of possibility, where collective attention on issues of equity and inclusion is perhaps higher than ever before. The work pushes against systems, structures and norms that are designed to be tenaciously exclusive. It involves risk‐taking and humility, where all people involved can learn from mistakes and embrace not knowing. We invite readers to remain grounded in the hopefulness of Freire and others who believed in more equitable futures, and to constantly ask themselves and others, in every possible interaction that they encounter in their sphere of influence, what practical steps they can take to cultivate meaningful inclusion in research ecosystems. This article offers practical steps for people situated in a variety of roles across research systems and settings, providing a beginning guide for creating more authentic inclusion.

## AUTHOR CONTRIBUTIONS

This manuscript involved collaborative and inclusive authorship. Lead author, Dr. Katrina Plamondon, facilitated and spearheaded writing, guided by the collaborative, dialogic efforts of this team with all authors making substantial contributions to the analysis and interpretation of data. All contributing authors are listed alphabetically after Katrina Plamondon, with each person contributing to critically reflective dialogues used to generate the content of this manuscript, including the discussion of results and recommendations. Katrina Plamondon, Davina Banner, Miranda A. Cary, Heather Gainforth, Alison Hoens, Anne Huisken, Nelly Oelke, Kathryn M. Sibley and Ursula Wick also contributed to the study conception and design. Katrina Plamondon, Miranda A. Cary, Melissa Faulkner, Kiranpreet Ghag, Anne Huisken, Damanpreet K. Kandola and Shaheer Khan contributed to data handling. All authors reviewed and revised drafts to the final version of this manuscript, and can comment on the accuracy and integrity of the work.

## CONFLICT OF INTEREST STATEMENT

The authors declare no conflict of interest.

## ETHICS STATEMENT

This study underwent ethical review at the University of British Columbia's Behavioural Research Ethics Board (REB# H18‐03416). In addition to ethical review of the research through this formal process, our team maintained an active commitment to relational ethics and equity‐centred principles and practices throughout the research process (discussed in the manuscript). All contributors to this study were provided detailed information about the research, its goals and procedures, and the ways in which privacy, confidentiality and contributions would be handled. All participants received copies of this information and provided consent before joining a deliberative dialogue.

## Data Availability

Data generated for this study are dialogic, with this manuscript providing the content and demonstrations of ideas, as chosen through a consensus‐based process. Given the nature of this data and our collaborative and iterative approach to analysis (and writing), the raw data are not publicly available. We are happy, however, to discuss and show more about our process and approach, using examples of how we move from dialogue transcript to consensus‐based decisions. The data that support the findings of this study are available from the corresponding author upon reasonable request.
