# Correction to “Relational Practices for Meaningful Inclusion in Health Research: Results of a Deliberative Dialogue Study”

**DOI:** 10.1111/hex.70056

**Published:** 2024-10-07

K. Plamondon, D. Banner, M. A. Cary, et al., “Relational Practices for Meaningful Inclusion in Research: Results of a Deliberative Dialogue Study,” *Health Expectations* (2023): 1‐15.

Coauthor Melissa Faulkner's name should be followed by “BSN” rather than “RN.”

We apologize for this error.

